# Emphysematous Hemorrhagic Cystitis Discovered During Laparoscopy for Suspected Bowel Perforation: A Case Report and Review of Diagnostic Strategies

**DOI:** 10.7759/cureus.69308

**Published:** 2024-09-13

**Authors:** Esteban Tapias, Donal Mor C O'Sullivan, Mohammad A Baig, Walker Creswell, Heather L Mateja, Penelope Mashburn

**Affiliations:** 1 General Surgery, Western Reserve Health Education/Northeast Ohio Medical University (NEOMED), Warren, USA; 2 General Surgery, Ross University School of Medicine, Bridgetown, BRB; 3 General Surgery, American University of Antigua College of Medicine, Osbourn, ATG

**Keywords:** bladder hemorrhage, chronic foley catheter, citrobacter brakkii, diabetes mellitus, diagnostic laparoscopy, emphysematous hemorrhagic cystitis (ehc), pelvic ct scan, rare urological conditions, urinary tract infection (uti), urine culture

## Abstract

Emphysematous hemorrhagic cystitis (EHC) represents an uncommon complicated urinary tract infection. The primary pathophysiology involves the inoculation of gas-producing bacteria or fungi in the bladder wall, leading to inflammation and ischemia. In this report, a case of EHC is presented, which was encountered in a 69-year-old male with multiple underlying comorbidities, highlighting the diagnostic challenges, clinical course, and management strategies employed. Through a review of the literature, the aim is to elucidate the patient presentation of EHC, emphasizing the importance of early recognition and multidisciplinary collaboration in optimizing patient outcomes. This case serves to contribute to the expanding body of knowledge surrounding this rare yet clinically significant condition, especially in at-risk populations.

## Introduction

Emphysematous hemorrhagic cystitis (EHC) is a rare form of complicated urinary tract infection (UTI) that can be caused by various gas-producing bacterial or fungal organisms [1]. The introduction of these microorganisms into the urinary tract, primarily *Escherichia coli*, *Klebsiella pneumoniae*, and *Clostridium perfringens*, serves as a cornerstone in the pathogenesis of EHC [2]. These organisms ferment urinary glucose, producing carbon dioxide and hydrogen gas, which accumulate within the bladder wall, leading to tissue ischemia, mucosal damage, and subsequent gross hematuria [3]. Rarely, the *Candida* genus of fungi can also cause EHC [1].

Clinical manifestations of EHC vary widely, ranging from mild urinary symptoms, including dysuria and hematuria, to severe, systemic manifestations, such as sepsis and renal failure [4]. Patients may initially present with nonspecific symptoms, which can obscure the diagnosis and delay appropriate intervention [4]. Moreover, the presence of gas within the bladder wall may not produce crepitus on physical examination, necessitating a high index of suspicion and judicious utilization of diagnostic imaging modalities such as a computed tomography (CT) scan [5].

Epidemiological data regarding its exact prevalence are limited, primarily due to its rarity and underreporting in the literature. However, emphysematous cystitis is more commonly observed in specific patient populations, such as individuals with diabetes mellitus, immunocompromised states, malignancy, or a history of bladder instrumentation, with diabetes being the strongest risk factor [6]. EHC is also rarer in the male diabetic population, with a female-to-male predominance of about 2.2:1 [2]. Here, we present a case of a diabetic male patient with a chronic Foley catheter and recurrent urinary tract infections who consequently developed EHC masked by possible viscus perforation.

## Case presentation

The patient is a 69-year-old Caucasian male with a past medical history of cerebrovascular accident, dementia, hypertension, diabetes mellitus type II, gastroesophageal reflux disease, atrial fibrillation treated with apixaban, recurrent urinary tract infections, benign prostatic hyperplasia with a chronic Foley catheter, and myocardial infarction with one stent placement presenting to the emergency department from a nursing care facility with altered mental status, recent multiple falls, and diarrhea. The patient’s Foley catheter was noted to be draining brown urine, and a physical examination revealed diffuse abdominal tenderness without rigidity or guarding. He was tachycardic (121 beats per minute), afebrile, and experienced one episode of coffee ground emesis. 

**Figure 1:**
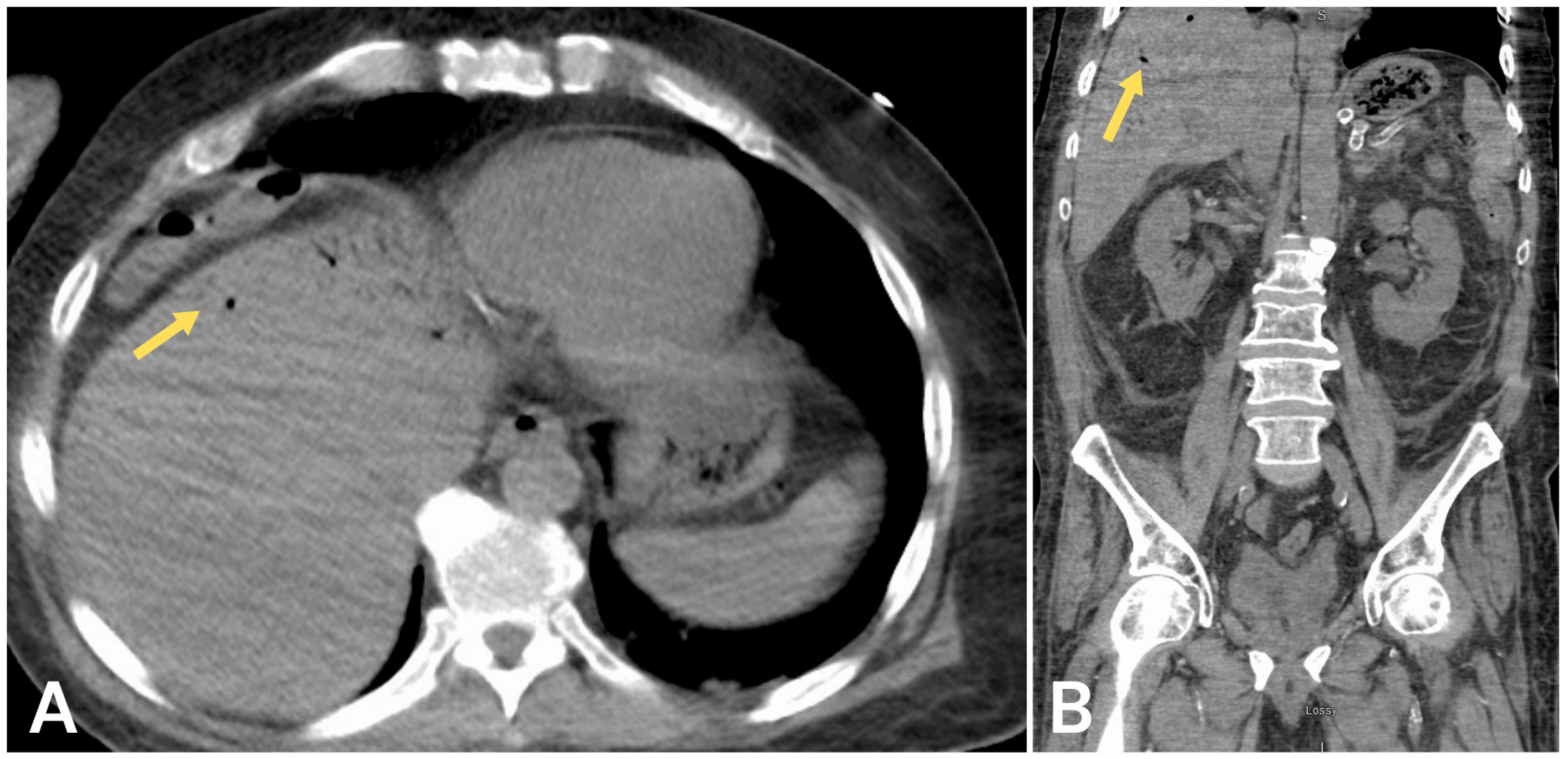
CT imaging revealing branching air/lucencies in the periphery of the liver parenchyma most consistent with portal venous air. Bowel ischemia is a common cause of portal venous air. (A) Axial view. (B) Coronal view.

Laboratory studies of the patient revealed leukocytosis with neutrophilic predominance, lactic acidosis, and elevated creatinine (Table 1). Urinalysis revealed numerous red blood cells with bacteria and yeast. A kidney-ureter-bladder (KUB) X-ray showed nonspecific bowel distention. CT imaging without contrast of the abdomen and pelvis showed abnormal lucencies within liver parenchyma most consistent with portal venous air (Figure 1), diffuse mesenteric edema and inflammation, bilateral hydroureteronephrosis, and soft tissue anasarca concerning a perforated viscus.

**Table 1:** Trend of select laboratory values BUN, blood urea nitrogen; eGFR, estimated glomerular filtration rate; Hgb, hemoglobin; WBC, white blood cell

Lab value	Reference range	On admission	Hospital day 3
Hematology			
WBC (×10^3^/mL)	3.5-10.5	17.1	8.0
Hgb (×10^3^/mL)	13.5-17.5	13.4	10.9
Platelets (×10^9^/L)	150-450	142	50
Neutrophils (%)	34-71	51	73
Band neutrophils (%)	0-8	35	21
Lymphocytes (%)	15-44	6	4
Monocytes (%)	0-10	1	2
Eosinophils (%)	0-7	0	0
Basophils (%)	0-2	0	0
Metabolic panel			
Sodium (mEq/L)	137-146	138	138
Potassium (mEq/L)	3.5-5.3	4.6	4.0
Chloride (mmol/L)	98-107	102	105
BUN (mg/dL)	5-25	69	74
Creatinine (mg/dL)	0.6-1.4	3.8	2.4
eGFR (mlL/min/1.73m^2^)	≥90	16	28
Glucose (mg/dL)	<100 fasting	323	139
Lactic acid (mmol/L)	0.5-2.0	5.2	1.6
Albumin (g/dL)	4.0-5.0	3.4	2.7

General surgery was consulted for further evaluation. On physical examination, the abdomen was soft and mildly distended, with moderate to severe tenderness to palpation through all quadrants, rebound tenderness, without guarding or rigidity. Due to the patient’s altered mental status, an appropriate timeline of the symptom onset and oscillations of severity were difficult to assess. It was decided to take the patient to the operating room for diagnostic laparoscopy, given the concerning findings.

**Figure 2:**
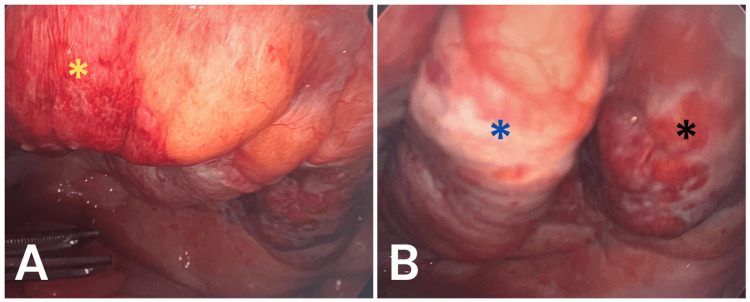
Intraoperative images revealing noticeably inflamed, erythematous, and firm median ligaments (blue) and medial ligaments (yellow and black) compared to surrounding tissue. (A) Right medial ligament inflammation (yellow). (B) Left medial ligament (black) and median ligament (blue) inflammation.

During the procedure, the small bowel was inspected from the terminal ileum to the ligament of Treitz, in which there were no signs of inflammation, perforation, or ischemia. When inspecting the pelvis, the bladder, median, and medial ligaments were noticeably inflamed, erythematous, and firm compared to the surrounding tissue (Figure 2). There was also an aspect of the rectum that was noted to have moderate inflammation. There was a moderate amount of fluid in the pelvis and right upper quadrants. This fluid was dark and murky, which was suctioned, and intraoperative cultures were obtained. Based on these intraoperative findings and the patient’s presentation, the diagnosis was determined to be emphysematous hemorrhagic cystitis.

**Figure 3:**
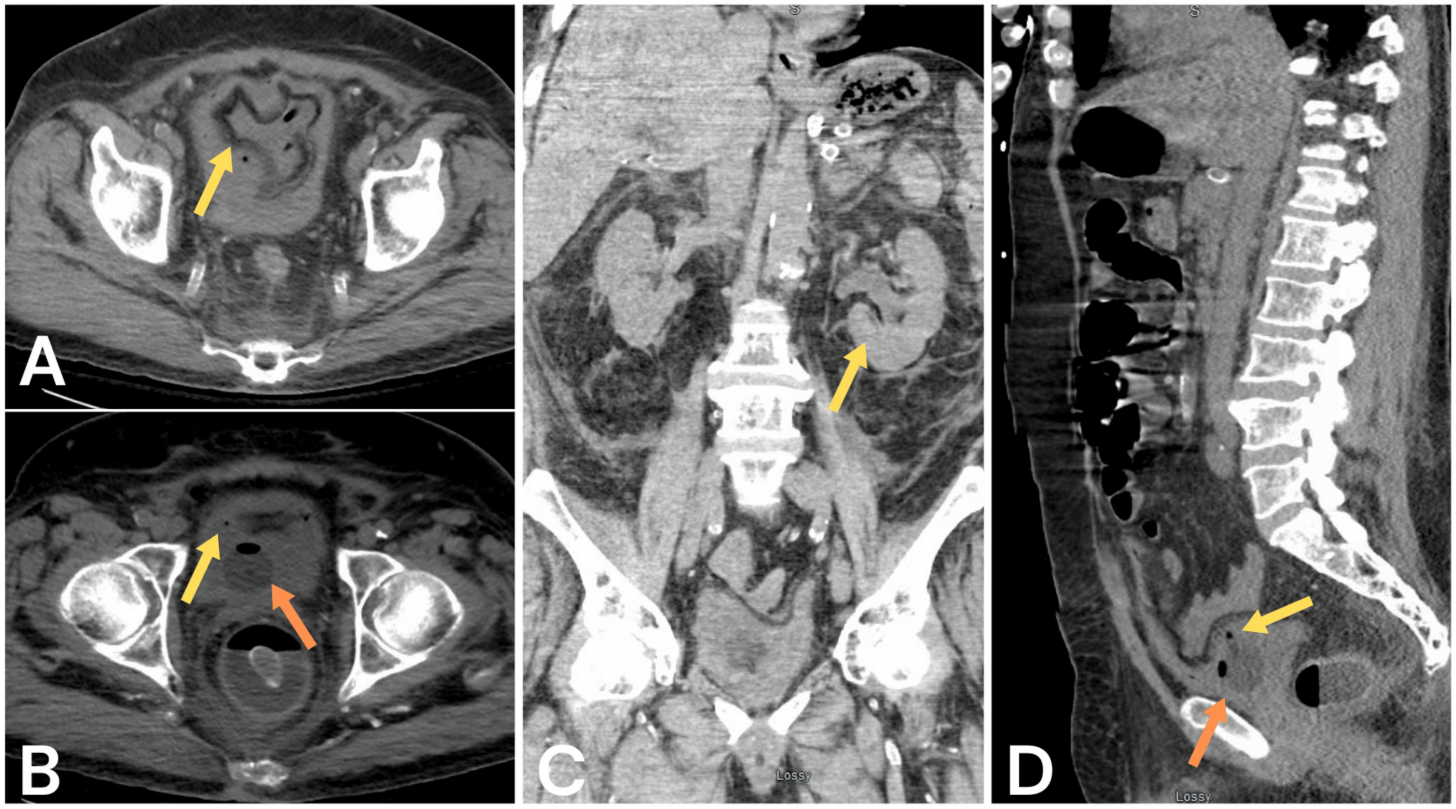
CT imaging without contrast demonstrating (A) axial view of irregularly shaped bladder with evidence of air in the wall, (B) axial view of air in the bladder wall (yellow) and with a Foley balloon in place (orange), (C) coronal view of hydroureteronephrosis of the left kidney, and (D) sagittal view of irregularly shaped bladder with air in the wall (yellow) and Foley balloon in place (orange).

Upon review of the CT imaging, there was no initial concern for air in the bladder wall, but in retrospect, the bladder was irregularly shaped, which may have concealed emphysematous areas within the bladder wall (Figure 3). The patient was admitted to the intensive care unit while infectious disease and urology were consulted. The urology team appreciated dark urine output from the Foley catheter at an acceptable rate and a down-trending creatinine. The urological team concurred with the diagnosis and deemed that no urgent urological intervention was necessary. They planned for a CT urogram and cystoscopy at a later date and recommended finasteride daily. 

Infectious disease began the patient on intravenous levofloxacin and cephazolin. After urine cultures grew predominantly *Citrobacter braakii*, they discontinued cephazolin. His recovery was complicated by sepsis, requiring the use of norepinephrine to maintain blood pressure for one day. Five days after admission, the patient was stable with a resolution of his acute kidney injury and discharged with the indwelling foley catheter to the nursing care facility with levofloxacin for 28 days and appointments to follow up in the outpatient setting. Upon a later admission, one month later, for a different reason, the patient still had an indwelling Foley catheter and was still experiencing altered mental status. However, the previous urinary symptoms had resolved with conservative management.

## Discussion

EHC is an uncommonly observed complication of a UTI, which is characterized by the presence of gas produced by bacteria or yeast inside the bladder wall [4]. The progressive inflammation will result in tissue damage of the bladder, causing hematuria, which classifies the diagnosis of EHC [3]. The etiology of this disease separates EHC from either simple bacterial cystitis (EC) [3]. In addition, EHC distinguishes itself from other forms of hemorrhagic cystitis (HC), which can be caused by various factors, including radiation therapy, chemotherapy (e.g., cyclophosphamide), and viral infections (e.g., adenovirus) [7,8]. An early diagnosis of this pathology is vital to improving prognosis and mitigating further morbid complications, including overwhelming infection, ascending extension to the ureter and kidneys, bladder rupture, and likely death [4,6]. It is documented that these complications can occur in up to 19% of cases, with an estimated mortality rate of approximately 7% [1,2,6]. Given the wide spectrum of clinical presentations, ranging from asymptomatic cases to severe infections and sepsis, clinicians should maintain a high level of suspicion when treating patients with a UTI who have risk factors for EHC, particularly diabetes mellitus [4]. It has been suggested that when treating a diabetic patient with a UTI, an abdominal radiograph should be taken to help rule out this complication [6]. 

While the presence of any specific symptom is not ubiquitous in all cases of EHC, most commonly, the patient will present with abdominal pain (80% of patients), gross hematuria (60% of cases), and ischuria (about 10% of cases) [1]. Pneumaturia is highly specific for this pathology, although it is difficult to detect without first being aware of EHC as a differential [1,4,6]. Frank peritoneal signs, as in our patient, are present in about 6.4% of cases [6]. Other symptoms often associated with acute cystitis (e.g., dysuria, urinary frequency, and urinary urgency) are observed in about 50% of cases of EHC, and if they do appear, they are typically quite mild [1,6]. Fever presents in about 30-50% of cases, which can serve as an alarm feature because it is often absent in acute cystitis [1].

When diagnosing EHC, imaging plays a crucial role in establishing a definitive diagnosis, as the diagnostic complexity of EHC lies in its ability to mimic other severe abdominal conditions [1]. Various modalities can reveal features indicative of pathology; however, plain radiographs and CT imaging are most commonly used in reaching a diagnosis [1,6]. A plain abdominal radiograph is the most frequently employed method [1], with radiographic findings for EHC often showing "curvilinear areas of increased radiolucency delineating the bladder wall, separated from posterior rectal gas" [6]. Nevertheless, CT is recognized as the most sensitive modality, making it essential for diagnosis [6]. While bladder ultrasonography and cystoscopy are not required for diagnosing EHC, they can provide additional details that help confirm the diagnosis [1]. Ultrasonography may show "diffuse wall thickening and focally hyperechoic regions with dirty acoustic shadowing," and cystoscopy, as planned by the urology team in the case discussed, can reveal a concurrent bladder outlet obstruction [1,6]. Magnetic resonance imaging (MRI) has not demonstrated specific compelling evidence in the diagnosis of EHC [6].

In our case, the diagnostic approach was complicated by several factors. First was the appearance of severe abdominal symptoms, which appeared to be a possible perforated viscus. Second, our patient was altered from his baseline mental status and unable to deliver a detailed history. Third, the irregularly shaped bladder presentation on CT initially concealed the presence of gas trapping within the bladder wall, with the presence of portal venous air. Considering these complications, it was deemed necessary to perform a diagnostic laparoscopy for further evaluation. The presence of gross hematuria, significant bladder wall inflammation, and the intraoperative findings of inflamed median and medial ligaments all pointed toward a severe, complicated infection, distinguishing EHC from the more common forms of either EC or HC. This severe clinical presentation was most likely exacerbated by the patient having multiple risk factors such as diabetes mellitus, recurrent UTIs, advanced age, and a chronic Foley catheter [1,4]. 

In a patient with suspected EHC, it is crucial to perform blood cultures, urinalysis, urine cultures, and urine gram stains to identify the causative organism and guide antibiotic selection [5,6]. Empiric antibiotic therapy can be informed by prior UTI cultures, if available [1]. In this case, levofloxacin and cephazolin were initially chosen, but cephazolin was discontinued once *Citrobacter braakii* was identified, with the infection ultimately controlled by levofloxacin alone. Common antibiotics for EHC include fluoroquinolones, ceftriaxone, carbapenems, or aminoglycosides, with therapy tailored based on culture results for optimal efficacy [1]. Treating the underlying infection usually resolves bladder hemorrhage associated with EHC [7]. Most cases respond well to conservative management, utilizing antibiotics as well as bladder drainage with a large-bore Foley catheter and saline irrigation, in addition to addressing predisposing factors like glycemic control [1]. Only about 10% of cases require both surgical and medical treatment, with surgery reserved for those unresponsive to medical management or with severe necrotizing infections [1,2]. Surgery is rarely needed unless there is a bladder outlet obstruction [4]. Surgical options, depending on infection severity, may include debridement, partial cystectomy, or total cystectomy. In this case, a diagnostic laparoscopy confirmed EHC, but the condition was successfully managed conservatively. 

The management approach, in this case, highlights the importance of a multidisciplinary team, including urology and infectious disease specialists, in optimizing patient outcomes. The decision-making process should be guided by the severity of the infection, the patient's response to initial treatments, and the presence of any complications that might necessitate surgical intervention. This case report contributes to the existing literature on diagnosing and managing EHC, a rare condition that can easily be overlooked due to its ability to mimic other severe abdominal conditions. By highlighting the clinical signs and imaging findings specific to EHC, this case underscores the necessity of considering EHC in the differential diagnosis when encountering similar presentations. Doing so could potentially prevent the need for invasive procedures like diagnostic laparoscopy, emphasizing the value of thorough imaging and early recognition in improving patient outcomes.

## Conclusions

EHC is a rare but serious condition that demands a high index of clinical suspicion for timely diagnosis and treatment. Early recognition is crucial, as the condition can rapidly progress to life-threatening complications due to bacterial infection, which can cause emphysema, bladder wall weakening, ulceration, and hemorrhage. This case underscores the importance of urine culture and pelvic CT scans as essential tools in the diagnostic process. Particular attention should be given to at-risk populations, including patients on Sodium-Glucose Transport Protein 2 (SGLT-2) inhibitors, those with chronic Foley catheters, and individuals with diabetes. This case also highlights the need for clinicians to consider EHC in differential diagnoses, as earlier inclusion and appropriate imaging may prevent invasive procedures like diagnostic laparoscopy. Future research should aim to enhance diagnostic testing for EHC and develop screening criteria to better identify patients at risk, ultimately improving outcomes for this rare and challenging condition.
